# Artificial Intelligence in Surveillance, Diagnosis, Drug Discovery and Vaccine Development against COVID-19

**DOI:** 10.3390/pathogens10081048

**Published:** 2021-08-18

**Authors:** Gunjan Arora, Jayadev Joshi, Rahul Shubhra Mandal, Nitisha Shrivastava, Richa Virmani, Tavpritesh Sethi

**Affiliations:** 1 Department of Internal Medicine, Yale University School of Medicine, New Haven, CT 06520, USA; 2 Genomic Medicine Institute, Lerner Research Institute, Cleveland Clinic, Cleveland, OH 44106, USA; jayadev.joshi12@gmail.com; 3 Department of Cancer Biology, Perelman School of Medicine, University of Pennsylvania, Philadelphia, PA 19104, USA; shubhra.rahul@gmail.com; 4 Department of Pathology, Albert Einstein College of Medicine/Montefiore Medical Center, Bronx, NY 10461, USA; jollynitisha@yahoo.com; 5 Confo Therapeutics, Technologiepark 94, 9052 Ghent, Belgium; virmani.richa@gmail.com; 6 Indraprastha Institute of Information Technology, New Delhi 110020, India; tavpriteshsethi@iiitd.ac.in

**Keywords:** COVID-19, machine learning, artificial intelligence, drug discovery, SARS-CoV-2, pandemic, diagnosis, prediction, surveillance, vaccine

## Abstract

As of August 6th, 2021, the World Health Organization has notified 200.8 million laboratory-confirmed infections and 4.26 million deaths from COVID-19, making it the worst pandemic since the 1918 flu. The main challenges in mitigating COVID-19 are effective vaccination, treatment, and agile containment strategies. In this review, we focus on the potential of Artificial Intelligence (AI) in COVID-19 surveillance, diagnosis, outcome prediction, drug discovery and vaccine development. With the help of big data, AI tries to mimic the cognitive capabilities of a human brain, such as problem-solving and learning abilities. Machine Learning (ML), a subset of AI, holds special promise for solving problems based on experiences gained from the curated data. Advances in AI methods have created an unprecedented opportunity for building agile surveillance systems using the deluge of real-time data generated within a short span of time. During the COVID-19 pandemic, many reports have discussed the utility of AI approaches in prioritization, delivery, surveillance, and supply chain of drugs, vaccines, and non-pharmaceutical interventions. This review will discuss the clinical utility of AI-based models and will also discuss limitations and challenges faced by AI systems, such as model generalizability, explainability, and trust as pillars for real-life deployment in healthcare.

## 1. Introduction

COVID-19, caused by severe acute respiratory syndrome coronavirus 2 (SARS-CoV-2), is the worst pandemic since the 1918 Spanish Flu. Within weeks of the first outbreak in December 2019 in the Wuhan city of China, the disease took epidemic proportions in China and other countries. On January 30th, 2020, COVID-19 was declared as a Public Health Emergency of International Concern, and subsequently, on March 11th, 2020, COVID-19 was declared a pandemic by the World Health Organization (WHO). The COVID-19 pandemic has resulted in a total of 200.8 million cases worldwide, with a reported 4.26 million deaths as of August 6th, 2021 [1]. Owing to its high transmissibility and death rate amongst elderly and immunocompromised individuals, the disease has become the latest foe to humankind. In terms of the total number of infections and mortality, the USA, India and Brazil are the most severely hit by COVID-19 [1]. Despite mass vaccination all over the world, COVID-19 still poses a threat to human lives and livelihood [2]. India’s recent COVID-19 crisis suggests that the pandemic is far from over, and new strategies are required for the surveillance, diagnosis and identification of therapeutic solutions against COVID-19. In this review, we will focus on the role of Artificial intelligence (AI) and machine learning (ML) tools in managing the COVID-19 pandemic.

### 1.1. Pathophysiology of COVID-19

SARS-CoV-2, which was first transmitted from animal-to-humans, is primarily spread by the droplet route but is also suspected to have airborne, contact, fomite, fecal-oral, bloodborne, and mother-to-child transmissions [3,4,5,6]. It is important to note that both symptomatic and asymptomatic subjects can transmit the SARS-CoV-2 virus through secretions such as saliva or respiratory droplets while talking, coughing, or sneezing [7,8,9,10,11]. When the respiratory droplets containing SARS-CoV-2 virus come in contact with a susceptible person’s mouth, nose, or eyes, it can result in COVID-19 infection. Transmission can also occur indirectly when a healthy individual comes in contact with a contaminated object or surface (fomite transmission) [12,13,14,15]. The SARS-CoV-2 infection mainly causes mild to severe respiratory disease that may lead to death in some cases, though many people infected with the virus do not develop any symptoms (asymptomatic) [16]. Symptoms such as fever, dry cough, and tiredness mark the beginning of the SARS-CoV-2 infection. The infection can also result in a variety of other symptoms, including loss of smell or taste, chest pain, sore throat, difficulty in breathing, chills, muscle aches, headache, vomiting, nausea, diarrhea, and a loss of appetite. The average incubation period for COVID-19 in patients ranges from 2 to 14 days [17,18,19]. Once in contact, the SARS-CoV-2 virus infects the lining of nasal, laryngeal, and lung mucosal membranes, thus producing a large number of virus particles that, in turn, activate the immune system and leads to the production of cytokines [20,21,22,23].

One of the most common COVID-19 manifestations is severe pneumonia, which may cause shortness of breath [20,24]. COVID-19 infection can be divided into three main phases [25,26,27]: the initial phase where SARS-CoV-2 replicates and symptoms are generally mild; this is followed by a phase where respiratory symptoms continue, and infection stimulates the adaptive immune system, which if remaining uncontrolled, leads to a third phase causing hyper-inflammation and death [25]. SARS-CoV-2 can also directly bind to other cells expressing ACE2 (angiotensin-converting enzyme 2), such as renal tubular cells and testicular cells, causing damage to the kidney and testicular tissue of the patients [28,29,30]. However, the evidence of COVID-19 affecting reproductive organs remains contentious and needs to be validated. COVID-19 also affects the digestive system, which leads to loss of appetite, diarrhea, vomiting, and abdominal pain [25,31,32]. The disease could also affect the liver leading to elevated levels of aspartate transaminase and alanine transaminase [33,34,35]. In the early stage of infection, antivirals (e.g., remdesivir, lopinavir) can provide some benefit in limiting the virus spread, while immunomodulatory therapies such as anti-interleukin (IL)-6 or corticosteroids may be helpful in the more advanced stages [36,37,38,39]. To date, there are no infallible druggable targets established to treat COVID-19 associated pathologies [40]. Efforts have been made to develop new drugs that target the SARS-CoV-2 virus and host surface receptor binding, regulating endocytosis machinery; viral replication, multiplication, and assembly; or targets that regulate host-associated innate immunity [41,42,43,44,45,46,47]. 

### 1.2. Advancement of Computational Methods to Combat COVID-19 Pandemic

In the last two decades, evolvement in computational approaches and modeling led to a paradigm shift in research methodologies related to infectious diseases [48,49,50,51,52,53,54]. Advancements in AI algorithms have helped to analyze a great volume of data and make meaningful predictions, conclusions, and automation [55]. AI is described as an effort to mimic the cognitive functions of a normal brain, such as problem solving and learning with the help of data [56]. The wide spectrum of AI in healthcare includes rule-based systems, classical ML and deep learning (DL). ML is a branch of AI that solves problems based on experiences gained from the curated data, usually referred to as training data, and makes predictions or decisions without any explicit instruction by the user [57]. Based on the input data, ML can be divided into two categories- supervised and unsupervised ML. The supervised ML model is constructed based on labeled data, known as training data, and this model is used to make predictions on new data. Conversely, in unsupervised ML, the supplied data are unlabeled and categorized without any previous knowledge [57]. Apart from the classical ML algorithms such as support vector machines, random forest classifiers, k-means and hierarchical clustering etc., recently, artificial neural networks (ANN) have become quite popular. ANNs are ML algorithms that mimic biological neural networks based on the mathematical structure to solve complex data-oriented problems [58]. Deep learning comprises advanced ANN-based ML algorithms in which multiple layers of processing units are used to deduce higher-level features from the data [59]. Most of the supervised ML algorithms can work with small datasets that are organized and labeled, whereas deep-learning programs can work with raw, unstructured data and require much larger volumes [60,61]. AI is being utilized in healthcare and biomedical research with a variety of tasks such as basic research, medicine, patient and disease management, image analysis and medical devices [62]. For example, IBM’s Watson for Oncology tool has convincingly predicted drugs for the treatment of cancer patients. Similarly, Microsoft’s Hanover Project proposes a personalized cancer treatment option based on AI [57,63]. Predicting drug toxicity using ML techniques has also gained popularity over the years [64]. After the onset of the COVID-19 pandemic, several efforts have been made to apply AI techniques on data such as CT scans, X-ray images, and cough sounds to follow infection [65,66,67]. Several studies have utilized omics data to find repurposed drug candidates for COVID-19 treatment [68,69]. In addition to this, data from social media, mobile phones and news articles have been utilized to track potential hotspots and community infections [70]. Usage of these big datasets requires a careful balance between public health and protecting data privacy [71].

**Figure 1:**
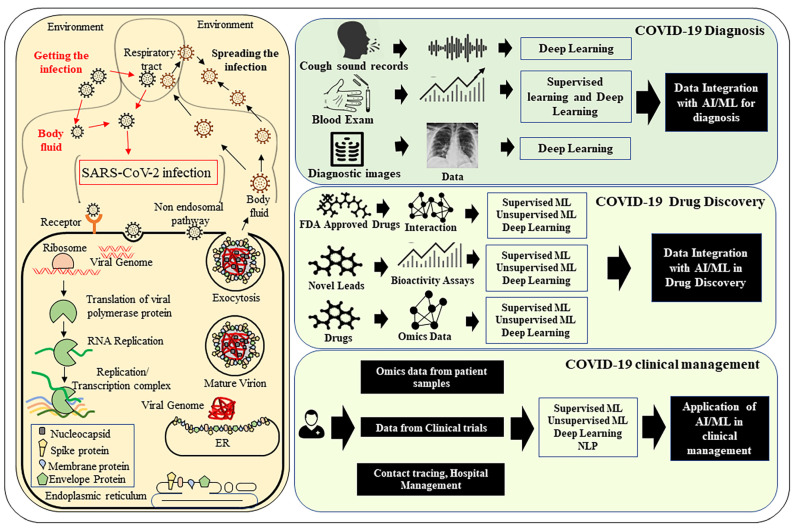
The application of Artificial Intelligence in handling COVID-19 pandemic. The life cycle of SARS-CoV-2 and COVID-19 disease etiology is shown on the left panel. On the right, examples of different applications of Artificial Intelligence (AI) are shown. AI-related tools can be useful in the accurate diagnosis of COVID-19 disease, finding new drugs, and analysis of data from clinical trials.

In this review, we will discuss how AI has been utilized during this pandemic to address key issues such as surveillance, detection, rapid diagnosis, drug discovery, and vaccine development (Figure 1). Furthermore, we will discuss several examples of ML and other AI applications that have been used previously in fighting complex diseases such as cancer and tuberculosis and can be applied in the case of COVID-19.

## 2. Application of AI in Surveillance of COVID-19

AI-based surveillance models can play a major role in predicting global infectious disease threats [72,73]. An integrated modeling approach that combines different types of individual data models such as travel data, mobile phone location tracking, epidemiological and behavioral pattern data is key to build a successful surveillance system [74]. This modeling approach requires an understanding of the target population at an individual level and, most importantly, during mass gatherings to restrict the spread of infection [75]. This type of integrated model-based platform could help identifying threats from infectious diseases of international concern as well as in anticipatory surveillance. Such integrated approaches also encourage mathematical modeling that can estimate the spread of infectious diseases with mass gatherings; simulate the effect of public health interventions aimed at the local and global level [76]. For example, a study predicted the infectious disease vulnerability index (IDVI) through an integrated modeling approach during the onset of coronavirus infection in Wuhan, China [77,78,79]. Multiple indicators such as travel information, country’s socio-economic condition, politics and health management facilities, and economic metrics are used to calculate IDVI scores [77,78,79,80]. IDVI scores range from 0 to 1, and a lower score signifies potential epidemic threats. Further, travel history along with common signs and symptoms through mobile phone-based online surveys can be used to build an AI-based model to predict risk factors [81]. 

**Figure 2:**
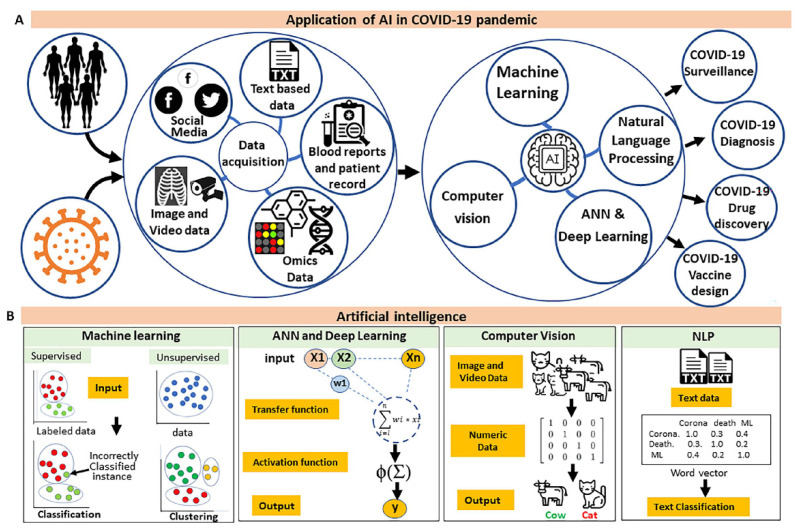
The role of AI tools in the COVID-19 pandemic. (**A**) The illustration depicts applications of ML and other AI tools in curated datasets from different paradigms to address the challenges associated with the COVID-19 pandemic. (**B**) An overview of existing AI techniques.

Different methods, such as multi-layered perceptron (MLP) and adaptive network-based fuzzy inference system (ANFIS), have been demonstrated to predict COVID-19 outbreak [82,83], whereas DL and other ML algorithms were favorable towards predicting COVID-19 spread in the future [84] (Figure 2). Alongside, mobile data-based surveillance, social networking data and associated public sentiment analysis are essential tools for the better management of the COVID-19 pandemic [85]. Contact tracing plays a major role in minimizing the spread of infection during any epidemic or pandemic. Mobile phone-based data are a great source to track individual activity, but it has to be wisely used as individual data privacy and security are always a concern. Different programs based on mobile applications, such as WeChat, have been developed, which utilizes smartphone-based GPS and social media data to perform contact tracing and risk assessment [86]. While such contact tracing may result in high false positives, a novel method has been proposed which uses data from six different smartphone sensors for contact tracing. This method outperforms other methods and identifies ~95% fewer false positives, reaching up to ~87% accuracy [87]. In Table S1, we have summarized some of the AI/ML models uses and their application in the surveillance of COVID-19.

In addition to contact tracing and predicting disease outbreaks, AI is also used in understanding COVID-19 risk assessment and public perception [88]. Combining data from different sources such as social media, demographic, mobility, and COVID-19 related epidemiological data, Ye et al. developed an AI-based program, α-Satellite. The development of α-Satellite required initial work on different tools that gather COVID-19 associated information from different sources in real-time and developed an attributed heterogeneous information network (AHIN) to use this data in a thorough manner. The key advantage of AHIN is that it can learn in a situation where data availability is restricted. Further, α-Satellite framework uses conditional generative adversarial net (cGAN) to create synthetic data and improve the AHIN. Finally, the α-Satellite uses a novel heterogeneous graph auto-encoder (GAE) to combine data from the close-by geographical areas and find the risk of any location. This program could be useful in assessing the risk at a community level in a hierarchical manner (geographical location such as state or country) [89]. The above applications of these powerful techniques suggest that the implementation of AI and ML models may provide a better prediction and management of pandemics in real-time [73,90,91]. 

## 3. Role of AI in the Screening of COVID-19 Infected Patients and Diagnosis 

The sudden increase in COVID-19 cases is imparting high pressure on healthcare services worldwide [92,93]. Precise diagnosis of COVID-19 infected patients is fundamental in the process of providing proper treatment and avoiding the overburdening of the healthcare system. Large-scale testing during a pandemic has been a challenge due to huge costs and a shortage of resources. Even the widely used RT–PCR test for the detection of COVID-19 positive (+) or COVID-19 negative (-) patient samples are not free from false-negative reports in low viral load conditions coupled with mild or no symptoms. Therefore, additional assistance from different AI-based modalities can be highly beneficial for accurate screening and diagnosis of COVID-19 and many other diseases [94,95] (Figure 1). A large number of symptom-based screening tools using decision rules can be thought of as one of the most common applications of AI. Further, we discuss some of the more advanced forms of AI, including ML and DL.

### 3.1. Imaging-Based Diagnostics

The various imaging techniques such as chest X-rays (CXR) and Computerized Tomography (CT) images are shown to be suitable in identifying COVID-19 (+) patients. However, the visual analysis of these images by a radiologist is subjective and therefore also prone to error. Researchers have shown that computer vision-based models can be accurate in analyzing these images [65,66,67]. Recently, an AI-based model has been developed which compared the performance between CT-based and CXR-based datasets [96]. Another study by Wang et al. showed that a convolutional neural network (CNN)-based model can be useful to identify COVID-19 infection in patients through CXR images [97]. A mathematical construct, CNN adaptively learns spatial hierarchies of data such as images [98]. The application of CNN-based deep learning methods in radiological image analysis for COVID-19 patients is discussed in great detail in a recent review [99]. A unified slice thickness is one of the limitations of the CT images, and generative adversarial networks-based AI models can overcome this challenge [100]. In another study, combining clinical and radiological imaging data with AI algorithms is shown to be more effective in identifying COVID-19 (+) patients than a senior thoracic radiologist [101]. An AI-based predictive diagnostic model was built based on chest CT findings with clinical symptoms, exposure history and laboratory testing data. This model identified COVID-19 infection with ~84.3% sensitivity and AUC (Area under the ROC Curve) of 0.92 [101]. Interestingly, researchers came up with a new set of descriptors based on the shape and texture of chest x-ray images in combination with a support vector machine (SVM) to differentiate COVID-19 from bacterial and viral pneumonia. This SVM-based model achieved ~89% accuracy and sensitivity while significantly lowering the computational cost as observed in DL-based methods [102]. In addition, Belfiore et al. projected the role of Thoracic VCAR (GE Healthcare, Milan, Italy), an AI-based software in COVID-19 diagnosis. The software is capable of doing automated lung segmentation and quantitative measurements to help in the assessment and follow-up of lung diseases [103,104]. Overall, these examples suggest that ML and other AI-based approaches can be useful in the objective assessment of imaging data obtained from COVID-19 patients.

### 3.2. Blood Analysis Tests

Routine blood exams provide various blood cell and other biochemical parameters that can be used for differential diagnosis. Generally, routine blood exams data in numerical form such as Whole Blood Cells count, blood sugar level, Hemoglobin, etc., can be used as a feature set to build classification and regression models. Combining blood tests with advanced AI-based methods can significantly improve the sensitivity and accuracy of diagnosis [105,106,107]. In the recent past, several studies have been published which show the applicability of these techniques in predicting common diseases [107,108]. Alsheref et al. assessed various ML algorithms to detect blood diseases. In this study, the author assessed the predictability of commonly used supervised algorithms to detect blood diseases, and they achieved ~98% accuracy to predict the occurrence of blood disease with LogitBoost algorithms [108]. Park et al. built three models, LightGBM and extreme gradient boosting (XGBoost) ML models and a DNN (deep neural network) based model on 5145 cases and 326686 laboratory tests [109]. The authors proposed that among the three models, the ensemble model showed 81% F1-score and ~92% prediction accuracy against the most common diseases [109]. Not only does this blood analysis detect the disease, but it can also tell about the severity of a disease. In another such example, Karahan et al. has proposed an ML model to detect disease severity in Chronic venous insufficiency (CVI), which is a progressive inflammatory disease. In this work, the author concluded that variation in fibrinogen and albumin levels can predict clinical class with ~75% sensitivity and disease severity with ~90% sensitivity in patients with CVI [110]. A data mining and a statistical analysis-based study was conducted by Zeng et al. on data from 3090 COVID-19 patients. These data were derived from a total of 15 studies showing variability in neutrophils and lymphocyte count. The ratio of the blood cells can be utilized to monitor the severity and progression of the disease [111]. Despite the ethical and commercial boundaries across the globe, researchers are trying hard to make such useful data available in the public domain. Aljame et al. have utilized open source data provided by Albert Einstein Hospital in Brazil. The ensemble model was built upon 5644 data samples to attain an outstanding performance with very high accuracy (~99.88%) and sensitivity (~98.72%) [112]. In one such study, two ML classification models were built based on hemato-chemical values from routine blood exams. This study was conducted on 279 patients with COVID-19 symptoms, and 177 were diagnosed as positive, while 102 as negative. ML models were able to predict positive and negative samples with high sensitivity (~95%) and accuracy (~86%). This study demonstrated the applicability and clinical usefulness of combining blood examinations with ML as an alternative to routine genomics-based approaches such as RT-PCR [113]. In another independent study, the researchers used random forest (RF), ANN, and a simple statistical test to diagnose SARS-CoV-2 in patients using full blood cell count data without knowing the symptoms or history of the patients. These techniques were able to diagnose SARS-CoV-2 among patients with a high accuracy range (AUC = 94–95%) from the community (AUC = 80–86%) [114]. Ko et al. have shown that these models can detect the risk of mortality and can be transformed into user-friendly and accessible open-source applications. The beatcovid web application can be utilized by any healthcare system for the management of COVID-19. The application can predict mortality with very high accuracy (~92%), specificity (~91%), and sensitivity (~100%) [115]. Despite the several successful trials and encouraging results, more effort is required to build a more generalized model based on robust datasets.

### 3.3. Analysis of Text and Voice Data 

In recent times, natural language processing (NLP) aims to develop computational algorithms to interpret human languages [116,117] (Figure 2). NLP and text mining have been adopted in medical research to extract and analyze data from various sources such as patient symptom records, sentiment data from social networking sites, and news articles to predict a medical condition or a disease outbreak. During the COVID-19 pandemic, efforts have been made to adopt such techniques to fight COVID-19 transmissibility. 

A recent study has used textual clinical reports to predict the occurrence of COVID-19 in patients. In this work, featured engineering algorithms, such as Bag of Words (BOW), report length, and Term Frequency/Inverse Document Frequency (TF/IDF), were applied to look for the best feature in the textual dataset. Selected features were used to train traditional and ensemble ML classifiers. The results suggested an outstanding accuracy of ~96.2% in detecting COVID-19 positive cases [118]. An AI-powered application, named AI4COVID-19, proposed that it is capable of diagnosing patients based on a sound recording of cough. This application communicates with the cloud and transfers the voice recording, and within seconds receives predictions based on the cloud-based AI engine [119].

## 4. Application of AI in Predicting COVID-19 Outcome 

While the accurate detection of SARS-CoV-2 in patients is the critical step towards treatment, a fast and early clinical assessment of the disease severity is also crucial to support decision making and logistical planning in healthcare systems [120,121,122]. Patients’ characteristics such as age, varied clinical symptoms, and comorbidities can help in categorizing the infection severity, need for hospitalization and predict the disease outcome [122,123]. Such prognosis-based prediction models for a given disease support the physician’s decision-making and assist in the screening of high-risk patients.

The mortality of COVID-19 patients can be potentially reduced by an early intervention, which is only possible by an accurate and early prediction of disease progression. XGBoost classifier, a high-performance ML algorithm, is used to identify three potential biomarkers; lymphocytes, Lactate dehydrogenase (LDH), and high-sensitivity C-reactive protein (hs-CRP). The XGBoost algorithm has great interpretability potential due to its recursive tree-based decision system and is shown to be ~90% accurate in predicting patient mortality approximately 2 weeks in advance [124]. Similarly, in another study, SARS-CoV-2 induced pneumonia was predicted based on seven laboratory parameters (prothrombin activity, urea, white blood cell, interleukin-2 receptor, indirect bilirubin, myoglobin, and fibrinogen degradation products) [125]. These parameters were identified by applying the least absolute shrinkage and selection operator (LASSO) logistic regression model based on features selected by the mRMR algorithm. This study showed that these multiple feature-based models can produce ~98% sensitivity and ~91% specificity in predicting SARS-CoV-2 pneumonia prognosis [125]. AI modalities can also help in predicting the personalized risk of adverse events or COVID-19 disease trajectory [126,127]. Further, different datasets such as patient health, travel history, geographical location, and demographic data were combined to build an AdaBoost Random Forest model. This model predicted the possible outcome of a COVID-19 patient with ~94% accuracy [128]. A study on a cohort of 13,690 patients has shown that the ML model can be applied effectively on a combined feature set. In this study, the patients’ clinical, demographic, and comorbidities data were analyzed to predict COVID-19 outcome, which helps the physician in decision-making [129]. Another example describes the better predictability of ventilation requirements for COVID-19 patients. This prediction uses ML models over physiological scoring based on modified early warning systems (MEWS). This model successfully predicted the need for a mechanical ventilator for a COVID-19 patient during hospitalization and helps in management of COVID-19 and improved patient care [130]. Another mortality prediction model for COVID-19 patients was built using the XGBoost algorithm based on clinical and demographic data. A combination of three main features, namely the type of patient encounter, minimum oxygen saturation, and age, showed high accuracy (AUC score of 0.91). This model can be easily implemented due to these three highly accessible clinical features pertaining to COVID-19 disease [131]. 

In Table S2, we have presented GitHub repository links from peer-reviewed literature that can be directly implemented in practice for COVID-19 diagnosis or disease outcome prediction to accelerate COVID-19 identification in patients, deciding proper treatment regime and possibly minimizing mortality.

## 5. Application of AI in Drug Discovery 

Antiviral agents and immunomodulators are the two major classes of compounds tested against COVID-19 [132,133]. Several repurposed drugs such as remdesivir, ivermectin, lopinavir, ritonavir, and other antiviral drugs emerged as somewhat effective treatment strategies for COVID-19 in the preliminary clinical studies [134]. So far, only a few drug candidates have looked promising as potential COVID-19 treatments [135]. AI algorithms enable the design of sophisticated and advanced drug development pipelines that can reduce the time and cost of the lengthy drug discovery process [136,137,138,139]. AI-based techniques are shown to be useful in the identification of repurposable drug candidates [69,140,141,142]. By applying various supervised ML and DL algorithms on experimental data, these techniques are proven to be more effective in identifying new antiviral drugs [143] (Figure 2). In a recent publication, Zeng et al. proposed that the AOPEDF (arbitrary-order proximity embedded deep forest approach) algorithm can predict novel drug-target interactions [144]. Based on a DL-based drug–target interaction model, Beck et al. predicted drugs that can target SARS-CoV-2-related proteins and are commercially available [145]. Pham et al. proposed DeepCE, a deep learning algorithm to repurpose drug compounds. The author demonstrated the application of DeepCE to predict potential leads for COVID-19 treatment [146]. In another study, an ML model was built to predict new indications for existing drugs and herbal compounds based on 1330 positive drug-disease associations though it was not directed against COVID-19 [147]. Overall, there is an enthusiasm for AI-based methods in finding repurposed drugs against SARS-CoV-2 [142,148,149]. Compounds with a potential likelihood of being a drug candidate demand sophisticated infrastructure and bioassay for the assessment of their toxicity, efficacy and response, interaction with other biomolecules, bioavailability, and metabolism [150,151]. The assessment of these pharmacokinetic properties of a drug candidate is considered a primary cause of failure of a drug during clinical trials [152]. For the SARS-CoV-2 drug discovery, an insight from the past studies that have combined cheminformatics and ML algorithms could be very useful [153]. ML learning algorithms are applied in the screening of millions of compounds against a druggable target in a very fast manner [154,155]. Using a similar method, Zhang et al. proposed a deep learning-based pipeline that is useful to screen peptides and small molecules against SARS-CoV-2 viral proteins [156]. The study used a densely fully connected neural network (DFCNN), which extracts more features from the data and allows faster virtual drug screening. To train DFCNN, the authors used the PDBBIND database, which renders structural information of proteins and macromolecular complexes [156]. In a recent study, Xu et al. used inhibitors of COVID-19 3CLpro and SARS 3CLpro proteins to build an ML-based model to predict novel inhibitors. Their training data set includes 66 active and 66 inactive compounds [157]. They employed six different ML classifiers (RF, SVM, LR, NB, DT, KNN) in their study. Based on probability (based on area under the ROC curve or AUC), the authors used Logistic Regression to screen the library. Using ML algorithms, Kabra et al. predicted antiviral peptides, which bind to SARS-CoV-2 protease [158]. ML algorithms used in the study allowed authors to work with SARS-CoV-2 virus sequences from different countries in a quick manner [158]. AI strategies developed here will not only be path breaking for COVID-19 drug discovery but also pave the way to develop new drugs against other infectious maladies [159,160,161,162]. We have tabulated the primary example of AI-based models used in COVID-19 drug discovery in Table S3.

In addition to this, ML-based methods can be effectively used in biomarker identification and drug sensitivity prediction that can improve clinical success rates [163,164]. With so many therapies emerging for COVID-19, AI-based tools can help in clinical trials andnovel treatments that are safe and effective (Figure 1). These algorithms can also be used to analyze the data from failed or suspended drug trials for COVID-19. Analysis of this observational evidence can be further used to assess uncertainty and generate causal inference to improve the design of future studies [55]. One way to improve the speed of clinical trials for drugs against COVID-19 is to avoid the traditional multi-phase route and design dynamic ML-based adaptive trials that start with a small group and continue into a trial-collection loop in which the collected data are used to determine pivot or continuation [30,165,166]. 

The above examples show several applications of ML and other AI techniques in drug target detection and assessing the impact of the mutation on existing targets, which can be utilized in the case of SARS-CoV-2 and associated pathologies (Figure 1). 

## 6. Application of AI in Vaccine Development and Delivery

One of the best possible strategies to combat COVID-19 is to develop a vaccine. Several virus components are used to develop an effective vaccine, namely the whole virus, the Spike (S) protein, Nucleocapsid (N) protein, and Membrane (M) protein [167,168,169,170]. Some of the vaccine candidates that got EUA approval during the COVID-19 pandemic, e.g., Comirnaty (Pfizer/BioNtech), mRNA-1273 (Moderna), Covishield (Oxford-AstraZeneca) and JNJ-78436735/Ad26.COV2.S (Johnson and Johnson), have been developed exploiting these viral components [171,172,173,174,175,176,177]. Though these vaccines are authorized by the the United States Food and Drug Administration (FDA), these possible interventions still have safety concerns and are less likely to give complete protection [178]. More so, side effects such as allergic reactions have been reported on the administration of these vaccines [179,180,181]. The challenges in manufacturing, storage, logistics, and issues related to the safety and efficacy of different vaccine candidates can be overcome by AI algorithms. As for any vaccine-induced immune response, the first step after COVID-19 vaccine administration is the presentation of antigenic peptides by major histocompatibility complex (MHC) class II molecules (or called Human Leukocyte Antigens) present on the surface of antigen-presenting cells. These exogenous peptides displayed by MHC class II molecules bind to the T-cell receptor of CD4^+^ T cells. Similarly, MHC class I molecules bind to CD8^+^ T-cells and activate the cytotoxic lymphocytes. Together, MHC-I and MHC-II molecules induce antigen-specific responses, which are central to vaccine-induced immunity. One of the most direct applications of ML and other AI-based strategies in vaccine development is to identify the presence of antigenic peptides presented by MHC-II. As an example, ML was used to develop programs such as MARIA (major histocompatibility complex analysis with recurrent integrated architecture) and MoDec that predicts antigen presentation [182,183,184]. Various AI-related tools have been used to analyze SARS-CoV-2 viral peptide presentation on MHC molecules from patients to understand natural immunity. Such an understanding may directly or indirectly help discover COVID-19 specific immune response and assist in designing an effective vaccine [185,186,187]. Ong et al. have used Vaxign-ML-based reverse vaccinology tools to predict targets that can be used to develop a safe and effective COVID-19 vaccine [169,188]. 

On the other hand, AI tools can help the local governments to assess public perception of COVID-19 vaccines and help in spreading vaccine awareness to the public. The main role of AI is basically to analyze all previous data and predict where the disease may progress in the future. This will not only help in analyzing but also understanding and suggesting paradigms for the development of future vaccines based on the number of cases studied, including confirmed, recovered, and patients who succumbed to the disease. The key advantages of AI are speed and accuracy with which it identifies these cases and its utility in screening for diagnosis and drug/vaccine development. Arshadi et al. developed Corona-DB-AI, a collection of compounds, peptides, and epitopes related to COVID-19 therapeutics. This dataset can be used for training models in order to extract COVID-19 treatment [189]. More recently, a study conducted at MIT’s computer science and AI lab has enlightened the use of AI in predicting its efficacy based on racial and minority populations [190,191]. The study has used two ML-based programs OptiMax and EvalMax, which work in tandem. OptimAX helps in the identification of the relevant peptide and designing peptide vaccine. EvalMax works with genetic structures of various racial ethnicities and finds which HLA (Human Leukocyte Antigen) haplotype frequencies work with specific peptides. The results with Optivax suggested that Spike protein of SARS-CoV-2 alone may not be effective in providing complete immunity to all the racial ethnicities. The study suggests that the addition of some peptides can enhance the immune response [190]. AI modalities are also used in effective vaccine design and assessing the safety of these vaccines [192,193,194].

## 7. Application of AI in Predicting Possible Viral Mutational Landscape 

High infectivity combined with a high mutation rate has made COVID-19 very challenging and deadly; thus, new SARS-CoV-2 infections are increasing unprecedentedly. [195]. Recent research based on AI has provided significant insight in predicting these mutational landscapes [196]. Hie et al. have developed an NLP-based algorithm that can predict mutations that have the potential to escape from the immune system and preserve the pathogenic capability of a virus strain. Using this model, authors are able to predict structural escape patterns of various viruses, including SARS-CoV-2 [197]. In a similar work, Salama et al. have presented a proof of concept by applying neural network and rough set technique on the genetic mutation prediction of Newcastle Disease Virus. The proposed technique verifies a correlation between the mutation of nucleotides and successfully predicts the nucleotides in the next generation [198]. Malone et al. used an AI-based algorithm to develop a broad-spectrum vaccine against COVID-19 that can provide maximum coverage for various COVID-19 strains. In this study, authors have evaluated around 3400 SARS-CoV-2 sequences that are used in the model for predicting epitope hotspots [199]. An recurrent neural network (RNN)-based Long Short-term Memory (LSTM) model has also shown very promising results in predicting the future rate of mutation in a person’s body after COVID-19 infection. Haimed et al. proposed a viral reverse engineering approach in which they try to find pattern similarity in viral protein and genomic sequences, and further mutational changes were extracted based on the phylogenetic tree to capture the evolutionary behavior [200]. Finally, a possible viral evolutional instance was predicted based on these two observations combined with the LSTM model [200]. The availability of several vaccines worldwide has created confidence among the community to tackle this challenge. However, the emergence of new and deadly strains, such as the B.1.617.2 (delta) variant, puts the healthcare system under pressure and uncertainty about the future efficacy of the available vaccines. Not only the prevention but the treatment of COVID-19 is also affected by new variants; hence, these methods have emerged as a key in tracking, predicting, and forecasting the mutational landscapes to manage COVID-19 disease [201,202]. 

## 8. Challenges and Limitations Associated with AI

AI is poised to play an increasingly important role in all areas of healthcare. However, the real-world scaling of such solutions poses many challenges and limitations. Validation, generalization, explainability, interpretability, risk mitigation, fairness, and inclusiveness are some of the key challenges in making AI-based decisions in medical and public health settings [203]. Generalization refers to the ability of AI-based algorithms to perform efficiently in different settings. Several concerns need to be addressed as the use of ML and other AI tools are increasing day to day in critical decision making [204]. In AI, generalizability usually attributes to the ability of an ML algorithm to be effective across a range of inputs and applications [205]. The narrow context models always have risks that they can fail at the broad level when applied with different datasets [206]. However, generalizability cannot be summarized by a universally agreed definition [207]. Owing to the participation of AI in different applications, it is critical to create and govern these techniques in a credible and fair manner. In clinical and health care setups, the absence of transparency within the models, the privacy of the patient data, and the safety and liability-related issues are major challenges in terms of ethical and regulatory aspects of AI [208]. AI governance deals with issues such as bias and lack of transparency by engaging different stakeholders. The prime focus of ethical governance should be on handling ethical issues involved in clinical operations such as fairness, transparency, and privacy [209,210]. Explainability and interpretability are two important factors that need governance to monitor and enhance AI algorithmic fairness, transparency, and accountability [210]. In addition to this, ethical auditing can examine the inputs and outputs of AI algorithms and models for bias and potential risks [211]. One of the drawbacks of AI-based models is that their real utility remains largely untested. For example, in the case of COVID-19 research, AI-based models are theoretical [212,213]. For instance, although minimum oxygen saturation was identified as an important mortality predictor, it needs to be modeled alongside the supplemental oxygen delivered, a piece of data that may be missing in many models.

However, most of these challenges are being proactively addressed by the AI researcher community. In the clinical settings, COVID-19 has triggered the need to go digital, improve data literacy and explore assistive algorithms. Grassroot-level applications of AI in addressing public health and the supply chain are also helping in connected care [214]. In the near future, some of the AI tools may be employed in the decision making in medical supplies, humanitarian aids, population risk assessment, and at a certain level, clinical care and treatment [126,214,215]. 

## 9. Discussion and Conclusions 

The world is going through another wave of COVID-19 infections. Worldwide, daily rates of new infections have jumped significantly since March 2020, with deaths rising—this horrid emergency is again putting strain on the heavily-burdened healthcare system throughout the world. To control the pandemic and related stress on healthcare, scientists are testing the applicability of AI strategies [71,216,217,218,219,220,221,222,223,224,225].

The computational approaches have proven very effective in basic research, diagnosis, and treatment to fight against infectious diseases [54,226,227,228,229,230,231,232,233]. AI-based approaches have emerged as a useful tool/method in surveillance, diagnostic and discovery of new therapeutics [139,187,224,234] (Figure 2). Combining a vast variety of data such as blood exams, clinical images, and recording of cough sound with advanced ML techniques provides a quick and reliable alternative for diagnosis and assessment of the disease severity. COVID-19 patients show symptoms such as fever, fatigue, muscle ache, cough and respiratory issues. Since clinicians cannot identify patients who succumb to the disease early on, the AI/ML tools are shown to be effective and helpful in making clinical decisions. From evaluating the safety and efficacy of therapeutics, to help with imaging data analysis or contact tracing, AI has provided novel solutions in the fight against COVID-19. For example, AI is helping overcome barriers between repurposed drugs, clinical testing of therapeutic strategies, and drug authorization [165,166,235,236]. The application of AI strategies in COVID-19 also faces certain challenges. To fully utilize these strategies, it is important to address issues related to data privacy, concerns on data collection and handling practices, and governmental oversight. Even in the pre-pandemic 2019, many experts believed that AI has the potential to revolutionize healthcare, and while the risk of algorithmic bias and data privacy concerns are very real, there is little question that AI has proven its utility in the fight against COVID-19.

## Data Availability

No new data were created or analyzed in this study. Data sharing is not applicable to this article.

## Supplementary Materials

The following are available online at https://www.mdpi.com/article/10.3390/pathogens10081048/s1, Table S1: AI application in Surveillance of COVID-19, Table S2: Ready-to-use AI-based models available in GitHub for diagnosing COVID-19 and predicting disease outcome, Table S3: The application of AI-based models used in COVID-19 drug discovery.
