# Supplementary material for: Artificial Intelligence in Surveillance, Diagnosis, Drug Discovery and Vaccine Development against COVID-19
Source: Pathogens. 2021 Aug 18;10(8):1048. doi: 10.3390/pathogens10081048 (PMC8399076; doi:10.3390/pathogens10081048)
Supplement: Supplementary file 1 [file pathogens-10-01048-s001.zip › pathogens-1273657-supplementary.pdf]

pathogens

MDPI

Supplementary Materials

Table S1. AI application in Surveillance of COVID-19.

Application	Model	Metric Used to Evaluate Performance	Reference
AI and ML application in Surveillance of COVID-19	AI based model	Not Applicable	Srinivasa Rao and Vazquez., 2020
	ANFIS model	Example Country: Italy<br>correlation coefficient (r) value 0.999<br>root mean square error (RMSE) value 190.81–188.18	Ardabili et al., 2020
	MLP model	Example<br>Country: Italy<br>correlation coefficient (r) value 0.999<br>RMSE value 189.76–320.93	Ardabili et al., 2020
	ANFIS Model	Example Country: Hungry<br>mean absolute percentage error (MAPE): 38.44-39.95<br>RMSE value 248.06–119.42	Pinter et al., 2020
	Deep neural network (DNN)	RMSE<br>Confirmed 163335.65<br>Deaths 8554.55<br>Recovered<br>25415.03	Punn et al., 2020

Table S2. Ready-to-use AI-based models available in GitHub for diagnosing COVID-19 and predicting disease outcome.

Model Type	Type of Data Retrieved from COVID-19 Positive Patients	Availability	Reference
Diagnosis of COVID-19	Age, sex, Chest CT images with clinical symptoms (fever, cough etc.), Temperature (°C), exposure history and routine blood work data.	https://github.com/howchihlee/COVID19_CT	(Mei et al., 2020a)
	Chest CT and X-ray images.	https://github.com/Chen-WWWeixiang/diagnosis_covid19	(Jin et al., 2020)
	Chest X-ray images.	https://github.com/Bio-medical-Computing-UFPE/Ikonos-X-Desktop	(Gomes et al., 2020)
	Chest CT (computed tomography) images.	https://github.com/wangshuocas/COVID-19	(Wang et al., 2020b)
COVID-19 outcome prediction	Data retrieved from Kaggle, containing geographical location, travel history, clinical, and demographic data.	https://github.com/Atharva-Peshkar/Covid-19-Patient-Health-Analytics	(Iwendi et al., 2020)
	Age, sex, ethnicity, encounter type (inpatient/other), body temperature, blood pressure, oxygen saturation, smoking habit, and any pre-existing disease.	https://github.com/SBCNY/Clinical-predictors-of-COVID-19-mortality	(Yadaw et al., 2020)
	Age, sex, epidemiological history, initial symptoms, outcomes (survival/mortality), laboratory test results.	https://github.com/HAIRLAB/Pre_Surv_COVID_19	(Yan et al., 2020)

Table S3. The application of AI-based models used in COVID-19 drug discovery.

Drug	Target	Algorithm	Other Details	Reference
Atazanavir	SARS-CoV-2 3C-like proteinase	Deep learning-based model -Molecule Transformer-Drug Target Interaction (MT-DTI)	Kd ~94.94 nM<br>May also act on RNA-dependent RNA polymerase and other SARS-CoV-2 replication proteins	Beck et al., 2020
Remdesivir	SARS-CoV-2 3C-like proteinase	MT-DTI	Kd ~113.13 nM	Beck et al., 2020
Efavirenz	SARS-CoV-2 3C-like proteinase	MT-DTI	Kd ~199.17 nM	Beck et al., 2020
Ritonavir	SARS-CoV-2 3C-like proteinase	MT-DTI	Kd ~204.05 nM	Beck et al., 2020
Elbasvir	NS5A inhibitor	DeepCE model	Approved drug for Hepatitis C	Pham et al., 2021
Pibrentasvir	NS5A inhibitor	DeepCE model	Approved drug for Hepatitis C	Pham et al., 2021
Velpatasvir	NS5A inhibitor	DeepCE model	Approved drug for Hepatitis C	Pham et al., 2021
Ruzasvir	NS5A inhibitor	DeepCE model	Investigational drug for Hepatitis C	Pham et al., 2021
Samatasvir	NS5A inhibitor	DeepCE model	Investigational drug for Hepatitis C	Pham et al., 2021
Odalasvir	NS5A inhibitor	DeepCE model	Investigational drug for Hepatitis C	Pham et al., 2021
Coblopasvir	NS5A inhibitor	DeepCE model	Investigational drug for Hepatitis C	Pham et al., 2021
Baloxavir Marboxil		DeepCE model	Approved drug for Influenza A and B	Pham et al., 2021
Metocurine		DeepCE model	Approved muscle relaxant	Pham et al., 2021
Dactinomycin		DeepCE model	Approved drug for Cancer	Pham et al.,2021
C998-0189	SARS-CoV-2 3C-like protease	Densely Fully Connected Neural Network (DFCNN) model	Compound from ChemDiv dataset<br>Vina score (kcal/mol) − 8.5	Zhang et al., 2020
C998-0948	SARS-CoV-2 3C-like protease	DFCNN model	Compound from ChemDiv dataset<br>Vina score (kcal/mol) − 7.7	Zhang et al., 2020
C998-1046	SARS-CoV-2 3C-like protease	DFCNN model	Compound from ChemDiv dataset<br>Vina score (kcal/mol) − 7.6	Zhang et al., 2020
Meglumine		DFCNN model	Targetmol-Approved Drug library candidate<br>DFCNN Score ≥ 0.999	Zhang et al., 2020
Vidarabine		DFCNN model	Targetmol-Approved Drug library candidate<br>DFCNN Score ≥ 0.999	Zhang et al., 2020
Ganciclovir		DFCNN model	Targetmol-Approved Drug library candidate<br>DFCNN Score ≥ 0.999	Zhang et al., 2020
Tripeptide (combination of isoleucine, lysine and proline)	SARS-CoV-2 3C-like protease	DFCNN model	DFCNN score ≥0.997	Zhang et al., 2020
